# Neurological Manifestation of Incretin-Based Therapies in Patients with Type 2 Diabetes: A Systematic Review and Network Meta-Analysis

**DOI:** 10.14336/AD.2019.0303

**Published:** 2019-12-01

**Authors:** Le Gao, Shuqing Yu, Andrea Cipriani, Shanshan Wu, Yi Huang, Zilu Zhang, Jun Yang, Yixin Sun, Zhirong Yang, Sanbao Chai, Yuan Zhang, Linong Ji, Siyan Zhan, Feng Sun

**Affiliations:** ^1^Department of Epidemiology and Biostatistics, School of Public Health, Peking University, Beijing, China.; ^2^Department of Psychiatry, University of Oxford, Oxford, OX3 7JX, UK.; ^3^National Clinical Research Center of Digestive Diseases, Beijing Friendship Hospital, Capital Medical University, Beijing, China.; ^4^Department of Mathematics and Statistics, University of Maryland Baltimore County, Baltimore, MD 21250, USA.; ^5^Harvard Medical School and Harvard Pilgrim Health Care Institute, Boston, MA 02215, USA.; ^6^Primary Care Unit, School of Clinical Medicine, University of Cambridge, Cambridge, CB1 8RN, UK.; ^7^Department of Endocrinology and Metabolism, Peking University International Hospital, Beijing, China.; ^8^Department of Clinical Epidemiology and Biostatistics, McMaster University, Hamilton, Ontario, Canada.; ^9^Department of Endocrinology and Metabolism, People’s Hospital, Peking University, Beijing, China.

**Keywords:** Incretin-based therapies, type 2 diabetes, network meta-analysis, dizziness, headache

## Abstract

As a new class of antidiabetic drug, incretin-based therapies, which include dipeptidyl peptidase-4 inhibitors (DPP-4Is) and glucagon-like peptide-1 receptor agonists (GLP-1 RAs), have raised concerns about symptoms of withdrawal in patients with type 2 diabetes mellitus (T2DM), such as dizziness and headache. To systematically evaluate whether incretin-based therapies may lead to dizziness and headache in patients with T2DM compared to other traditional antidiabetic drugs or placebo. We searched Medline, Embase, the Cochrane library, and clinicaltrials.gov from inception through June 23, 2017, to identify randomized controlled trials of the safety of DPP-4Is or GLP-1 RAs versus placebo or other antidiabetic drugs in T2DM patients. We used the network meta-analysis under the frequentist framework to compare the association between multiple antidiabetic drugs and dizziness and headache. A total of 233 clinical trials with nine treatments and 147,710 patients were included: two incretin-based therapies, one placebo, and six traditional antidiabetic drugs (metformin, insulin, sulfonylurea, thiazolidinediones, alpha-glucosidase inhibitor, and sodium-glucose co-transporter 2). Compared to insulin, thiazolidinediones, or placebo, GLP-1 RAs statistically significantly increased the risk of dizziness (odds ratios [ORs]: 1.92, 1.57, and 1.40, respectively) and headache (ORs: 1.34, 1.41, and 1.18, respectively). DPP-4Is increased the risk of headache (OR: 1.22, 95% confidence interval [CI]: 1.02 to 1.46; moderate quality) and dizziness (OR: 1.46, 95% CI: 1.05 to 2.03; moderate quality) compared to insulin. Of the incretin-based therapies, DPP-4Is had a lower risk of dizziness than GLP-1 RAs (OR: 0.76, 95% CI: 0.67 to 0.87; high quality). Ranking probability analysis indicated that GLP-1 RAs may have the greatest risk of both dizziness and headache among the nine treatments (22.5% and 23.4%, respectively), whereas DPP-4Is were in the middle (46.2% and 45.0%, respectively). Incretin-based therapies increase the risk of dizziness and headache compared to insulin, thiazolidinediones, and placebo.

Review

Patient compliance with taking antidiabetic agents ranges from about 40% to 60% [1-3]; one study even found a maximal adherence rate of only 1% [4]. Many factors are related to poor compliance, including patient knowledge, patient beliefs, drug type, and drug side effects; of these factors, side effects contribute most to nonadherence [5]. Poor compliance may result in inadequate control of blood glucose.

Dipeptidyl peptidase-4 inhibitors (DPP-4Is) and glucagon-like peptide-1 receptor agonists (GLP-1 RAs) are incretin-based therapies, a new class of antidiabetic treatment. GLP-1 RAs are receptors of GLP-1, an incretin that can decrease blood sugar levels by enhancing the secretion of insulin [6, 7]. Because GLP-1 can be rapidly degraded by DPP-4 with a half-life of about 2 min, DPP-4Is can increase GLP-1 activity [6, 8, 9]. Although some of the benefits of this novel class of antidiabetic drugs including insulinotropic effects, low rates of hypoglycemia, no weight gain and improvement in β-cell function have been studied, compliance rates are unsatisfactory [10, 11]. The increasing popularity of incretin-based therapies in recent years has raised more and more concerns about safety [8, 12-14]. Multiple clinical trials have shown that incretin-based therapies may harm the central nervous system [9, 15]. Frequent dizziness and headache, symptoms of diabetes itself [16-19], may reduce patients’ compliance and worsen their glycemic control. These safety issues motivated the present study.

The objective of our network meta-analysis (NMA) was to evaluate the neurological safety of incretin-based therapies versus traditional antidiabetic drugs or placebo, particularly in terms of dizziness and headache, in patients with type 2 diabetes mellitus (T2DM) to enable practitioners to better manage adverse events to improve blood glucose control.

To date, five DPP-4Is (sitagliptin, vildagliptin, saxagliptin, linagliptin, and alogliptin) and five GLP-1 RAs (exenatide, liraglutide, albiglutide, lixisenatide, and dulaglutide) have been approved for use in health care [20].

## MATERIALS AND METHODS

This study was registered with the International Prospective Register of Systematic Reviews (CRD420 18091035). We reported this NMA according to the PRISMA for Network Meta-Analyses.

### Data sources and searches

Medline, Embase, the Cochrane Library, and clinicaltrials.gov were searched from inception through June 23, 2017, to identify both published and unpublished trials. We searched the databases using *glp-1 receptor agonists* and *dpp-4 inhibitors* as keywords or mesh terms accompanied by relevant free words. Example of the search strategy in Embase is provided in Supplementary Table 1.

### Eligibility criteria

We included only randomized controlled trials (either open-label, single-blind, double-blind, triple-blind, or quadruple-blind trials) published in English with available data on relevant outcomes in which incretin-based therapies and placebo or other antidiabetic drugs were compared. The adverse events included dizziness and headache, both from secondary outcomes.

### Study selection

All titles and abstracts were examined for inclusion by one senior reviewer (FS). Works that clearly did not meet the inclusion criteria (e.g., no T2DM, use of the same incretin-based therapies in both arms) were excluded. Then the full texts of all remaining articles were examined by two reviewers (LG and JY). Disagreements were resolved through discussion between the two independent reviewers or by the senior investigator (FS).

### Data extraction and quality assessment

ADDIS 1.16.5 was used to manage information extracted from trials, including study characteristics (author, publication year, duration of follow-up), participant details (age, sex, baseline treatment, duration of T2DM, baseline HbA1c), and reported outcomes in the experimental and control groups (number of events of dizziness and headache). Data extraction was performed by four investigators (LG, JY, SW, SY) independently and checked at random by one reviewer (LG). The risk of bias in each included study was independently assessed by one reviewer (LG) and then checked by another reviewer (SW) using the Cochrane Risk of Bias tool [21]. In addition, we used the Grading of Recommendations Assessment, Development, and Evaluation (GRADE) framework to assess the quality of each mixed comparison and the total ranking of treatments [21, 22].

### Data analysis

Direct comparison: We calculated pooled odds ratios (ORs) and 95% confidence intervals (95% CIs) of events of dizziness and headache using the random-effects model in STATA 13.1. *I*^2^ was used to describe the heterogeneity between pairwise comparisons. We included only double-blind, triple-blind, and quadruple-blind trials in the sensitivity analysis.

Indirect and mixed comparison: We used the frequentist framework to perform random-effects NMA. Pooled ORs and 95% CIs were also summarized. Next, we estimated the ranking probabilities of each treatment and then ranked the drugs using surface under the cumulative ranking curves. This procedure gives a percentage, interpreted as the probability of a treatment being the most effective without uncertainty on dizziness or headache events; 100% means that the treatment is certain to be the best and 0% means it is certain to be the worst. We also used the contribution plot to measure the percent contribution of each direct comparison to the mixed estimates, the indirect estimates, and the entire network. To assess publication bias, we used the comparison-adjusted funnel plot to detect the effects of small studies. Finally, we performed subgroup analysis (by age group, duration of T2DM, HbA1c% level, trial duration, sample size, and sponsorship), sensitivity analysis (excluding open-label trials), as well as univariate and multivariate meta-regression (by age, body mass index, HbA1c%, and duration of T2DM). All of these analyses were performed in STATA 13.1.

### Examination of assumptions in the NMA

To check the consistency of the NMA, we used the node-splitting model [23] to assess the inconsistency between direct and indirect treatment effects. Then we used the loop-specific approach to identify all triangular or quadrilateral loops in the network and estimate the respective inconsistency factors and their uncertainty [24]. As for heterogeneity, the predictive interval plot was used to estimate effect sizes and their uncertainty for all comparisons. In addition, the R 3.3.3 *netmeta* package was used to calculate the global *I*^2^ statistic.

**Figure 1.**
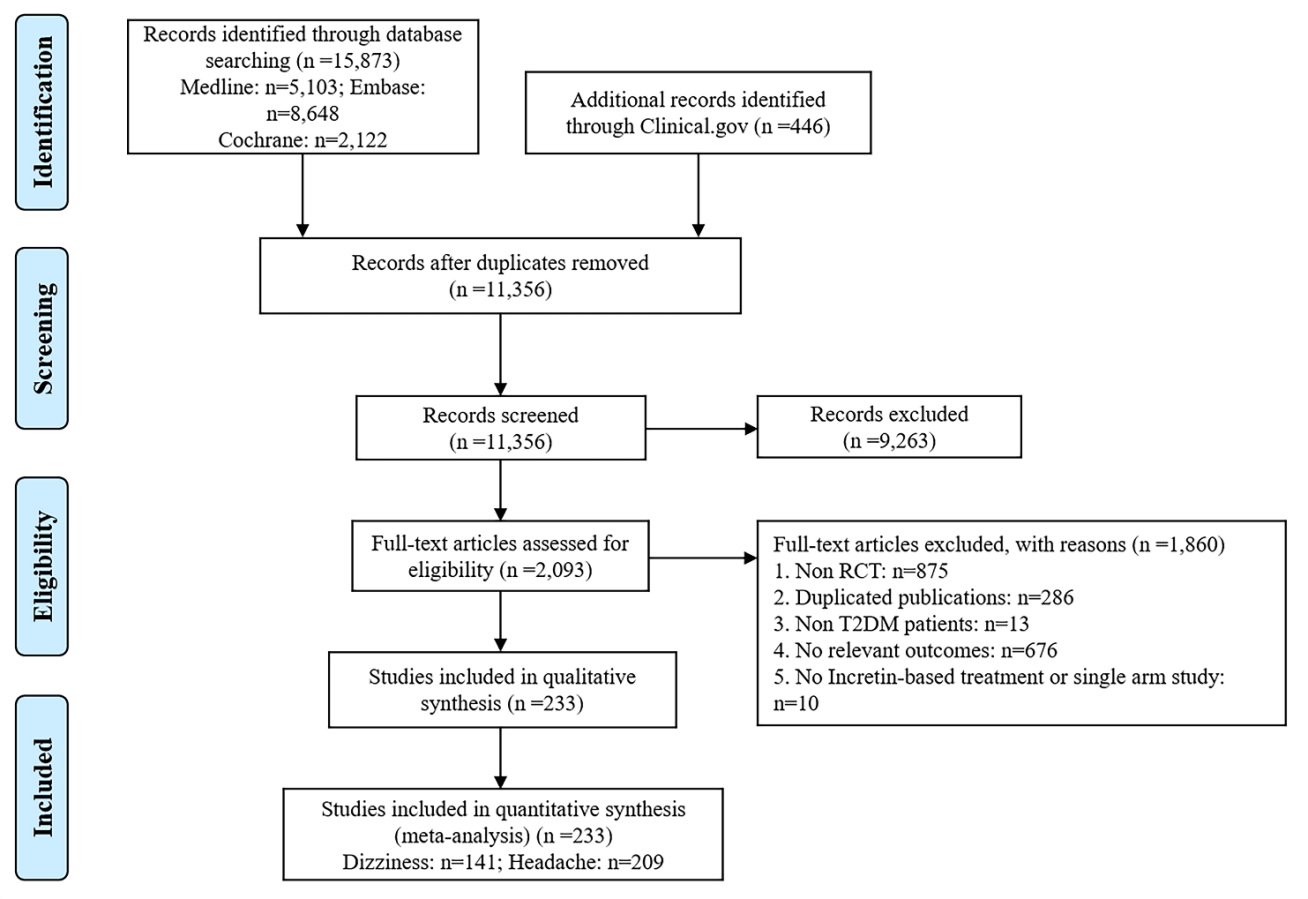
Flow chart of studies considered for inclusion.

## RESULTS

### Characteristics of the studies

A total of 233 trials with 147,710 participants met the inclusion criteria, of which 141 trials reported dizziness and 209 trials reported headache (Supplementary Table 2). These trials were published between 2004 and 2017, and the median trial duration was 26 weeks. Participants’ mean age was 56.32 years, the mean baseline HbA1c was 8.18%, and the median duration of T2DM was 6.4 years. Fig. 1 shows the flowchart of trial selection. The trials analyzed nine treatments: two incretin-based therapies (10 different DPP-4Is and 8 different GLP-1 RAs), placebo, and six traditional antidiabetic drugs (metformin, insulin, sulfonylurea, thiazolidinediones [TZD], alpha-glucosidase inhibitor, and sodium-glucose co-transporter 2). The network plots are shown in Supplementary Fig. 1. A total of 128 trials referred to DPP-4Is, 93 to GLP-RAs, and 12 to both DPP-4Is and GLP-RAs. Most of trials (95.3%) were two-arm studies, the other were three-arm (3.8%) and four-arm (0.9%) studies.

### Results of pairwise meta-analysis

**Figure 2.**
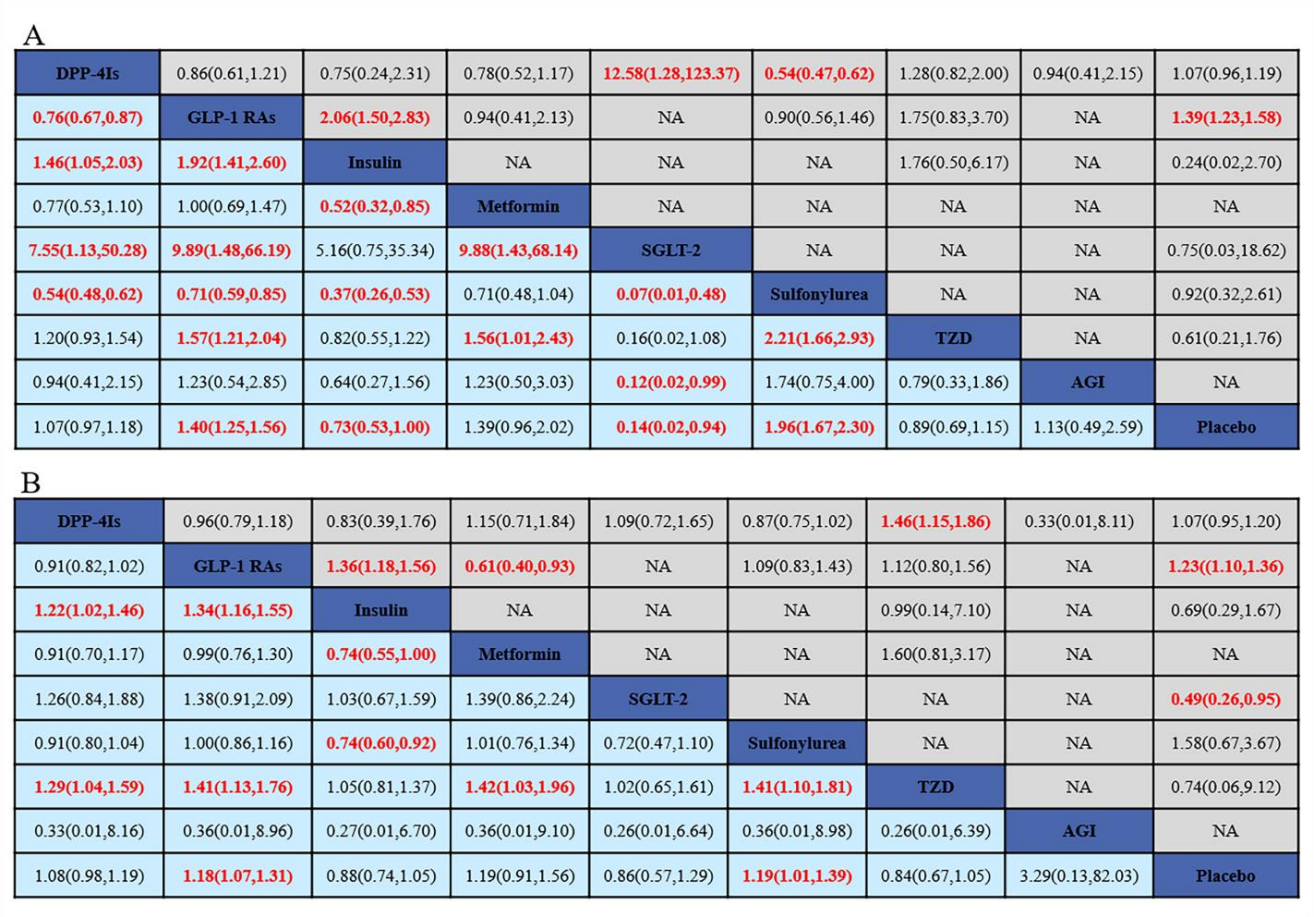
Odds ratios (ORs) with 95%CIs of NMA. For dizziness (A) and headache (B), results of direct comparisons were listed in the upper triangle, and the estimation was calculated as the row-defining treatment compared with the column-defining treatment. Results of NMA were listed in the lower triangle, the estimation was calculated as the column-defining treatment compared with the row-defining treatment. The statistically significant results were bolded in red. NA: not available. DPP-4Is: dipeptidyl peptidase-4 inhibitors; GLP-1 RAs: glucagon-like peptide-1 receptor agonists; SGLT-2: sodium-glucose co-transporter 2; TZD: thiazolidinediones; AGI: alpha-glucosidase inhibitor.

Fig. 2 shows the associations between incretin-based therapies and other active antidiabetic drugs and dizziness and headache according to pairwise meta-analysis. As for dizziness, DPP-4Is reduced the risk of dizziness versus sulfonylurea. Although DPP-4Is had a statistically significant effect compared to sodium-glucose co-transporter 2, the CI was broad because of the small sample size. Compared to insulin and placebo, GLP-1 RAs increased the risk of dizziness, with ORs of 2.06 and 1.39. As for headache, DPP-4Is increased the risk of headache compared to TZD. GLP-1 RAs had a more harmful effect than insulin and placebo. In contrast, compared to metformin, GLP-1 RAs had a protective effect, with an OR of 0.61.

### Results of the NMA

**Figure 3.**
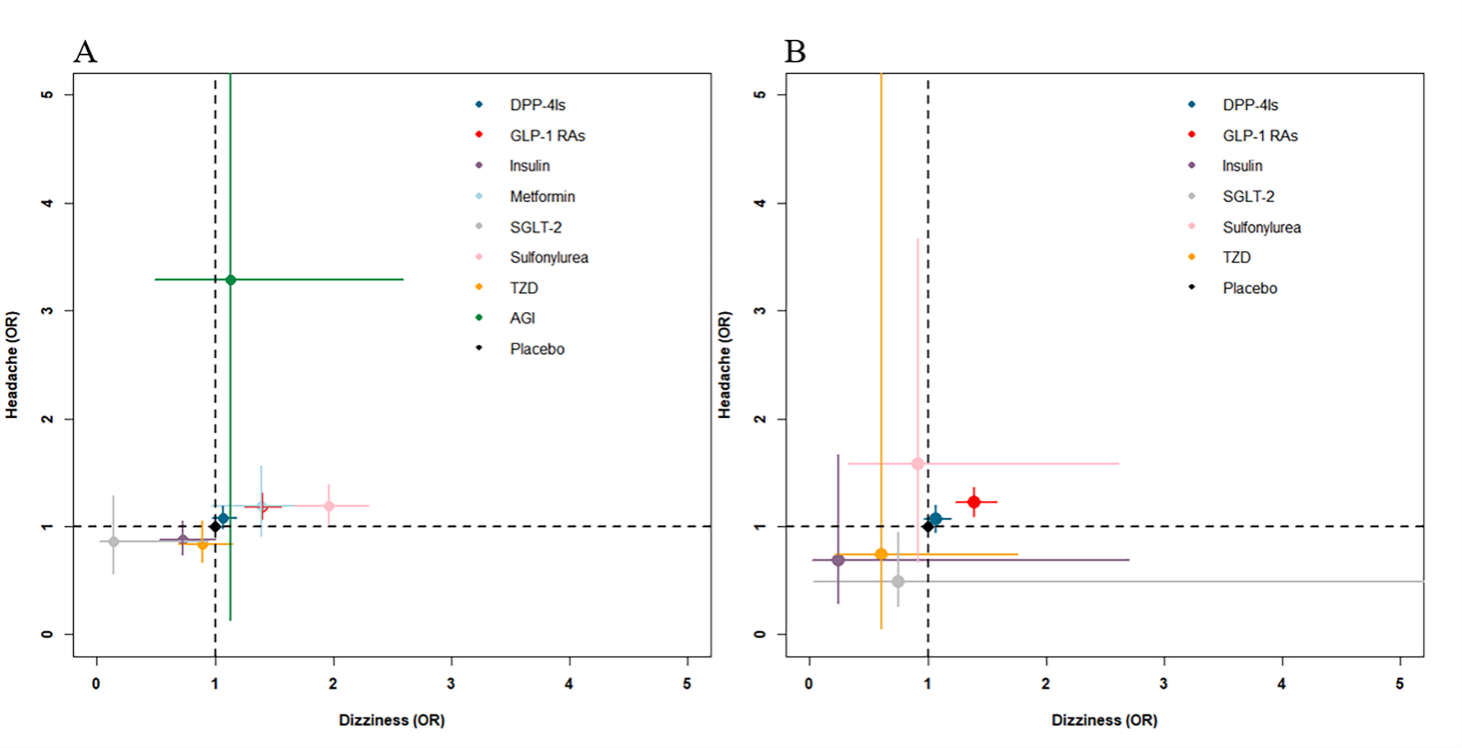
Two-dimensional graphs about risk on dizziness and headache. ORs in comparison with placebo (reference) of NMA (A) and direct comparisons (B) were used. Error bars are 95% CIs. Different drugs are represented by di?erent colored nodes. Metformin and AGI were not included in direct comparisons because no trials focus on these drugs compared with placebo. DPP-4Is: dipeptidyl peptidase-4 inhibitors; GLP-1 RAs: glucagon-like peptide-1 receptor agonists; SGLT-2: sodium-glucose co-transporter 2; TZD: thiazolidinediones; AGI: alpha-glucosidase inhibitor.

Results of the NMA are shown in Fig. 2. As for dizziness, the mixed effect was statistically significant for GLP-1 RAs versus insulin, TZD, and placebo with ORs of 1.92, 1.57 and 1.40, respectively. Meanwhile, DPP-4Is had a more harmful effect than insulin (OR=1.46, 95% CI: 1.05, 2.03). However, compared to sulfonylurea, DPP-4Is and GLP-1 RAs had a protective effect, with ORs of 0.54 and 0.71. In addition, DPP-4Is had a lower risk of dizziness than GLP-1 RAs (OR: 0.76, 95% CI: 0.67, 0.87). As for headache, DPP-4Is increased the risk of headache compared to insulin, TZD, and placebo (OR=1.22, 1.29 and 1.08, respectively). GLP-1 RAs had a similar effect, with ORs ranging from 1.18 to 1.41. Fig. 3 visually shows two-dimensional graphs for dizziness and headache in NMA and direct comparisons.

Supplementary Table 3 shows the ranking probability of the safety in terms of dizziness and headache. DPP-4Is ranked in the middle (ranked fifth for both dizziness and headache) and GLP-1 RAs ranked lower (ranked eighth for dizziness, ninth for headache) among the nine antidiabetic drugs, so did the comprehensive rankings of these two treatments in total safety for these symptoms. According to the contribution plot for the incretin-based regimens network of dizziness and headache (Supplementary Fig. 2), DPP-4Is and GLP-1 RAs versus placebo contributed the most.

### Methodological quality and risk of bias

Supplementary Fig. 3 presents the methodological quality of different outcomes. Of the total 233 studies included in our analysis, the large majority reported the use of random sequence generation (94.4%), allocation concealment (81.9%), blinding of participants and personnel (80.2%), blinding of outcome assessment (77.6%), complete outcome data (94.8%), and selective reporting (91.8%). In addition, only 5.2% of the trials were conducted by research institutions.

According to visual inspection of the funnel plot, publication bias was found for dizziness. In contrast, the funnel plot for headache was quite symmetric, which suggested no publication bias (Supplementary Fig. 4).

GRADE showed that the ranking of treatments ranged from very low to high, and for both headache and dizziness most comparisons were rated high or moderate (Supplementary Table 4).

### Results of assumptions in the NMA

Evaluation of the local inconsistency of dizziness and headache showed that most loops were consistent according to the CIs (Supplementary Fig. 5). Evaluation of the inconsistency by the node-splitting model did not reveal any significant difference in dizziness between direct and indirect comparisons, and only two comparisons (DPP-4Is vs. metformin and GLP-1 RAs vs. metformin) showed a significant difference for headache (Supplementary Table 5). This may have been because of different values for baseline HbA1c. The mixed comparison of DPP-4Is versus metformin came mainly from direct comparisons of DPP-4Is versus metformin and GLP-1 RAs versus metformin, as did the mixed comparison of GLP-1 RAs versus metformin. Moreover, from the distribution of several baseline factors, including age, body mass index, duration of T2DM, and HbA1c, we could see that baseline HbA1c differed significantly, which may have affected the transitivity of the results (Supplementary Fig. 6). It was also demonstrated in the subgroup analysis (Supplementary Table 6). In addition, the duration of T2DM and trial duration are likely other reasons for some of the inconsistency between direct and indirect comparisons (Supplementary Table 6).

Predictive intervals indicated that no comparisons were affected by estimated heterogeneity for dizziness, and 8.3% of the comparisons related to concerned drugs for headache had been slightly affected (Supplementary Fig. 7), which may be due to the difference of some baseline characteristics (Supplementary Table 7) and study design (Supplementary Fig. 8).

Testing for global inconsistency did not reveal any significant difference between consistency and inconsistency models (P=0.285 for dizziness and P=0.216 for headache). The global *I*^2^ was 0% (dizziness) and 6.7% (headache).

### Results of other analyses

Subgroup analysis: According to the subgroup NMA, compared to placebo, GLP-1 RAs had a more harmful effect on dizziness in patients with T2DM duration ≥ 5 years, mean HbA1c ≥ 7.5%, and trial duration ≤ 24 weeks. The results were roughly similar for headache (Supplementary Table 8).

Sensitivity analysis: Results of the sensitivity NMA with double-blind, triple-blind, and quadruple-blind studies only were generally consistent with previous studies for dizziness (Supplementary Fig. 9). For headache, results for GLP-1 RAs versus TZD and placebo were in line with previous studies, whereas other comparisons that included incretin-based therapies did not differ significantly.

Meta-regression: Univariate meta-regression showed that for headache, the pooled OR of DPP-4Is decreased by 0.96 for every 1-year change in duration of T2DM compared to placebo. Multivariate meta-regression showed similar results (Supplementary Table 9).

## DISCUSSION

With the increasing use of DPP-4Is and GLP-1 RAs, more and more people have begun to be concerned about the safety of these treatments. In recent years, there have been systematic reviews of the cardiovascular effects of incretin-based therapies [13, 25] and their risks of bone fractures [26, 27], respiratory tract infections [28], and pancreatic cancer [29]. Although some studies have reported dizziness and headache after taking DPP-4Is or GLP-1 RAs, these symptoms have usually been considered secondary outcomes and have not been taken seriously by doctors. In addition, we found two large cohort studies with sample sizes of more than 1,000, GLP-1 RAs were associated with a higher risk of dizziness than DPP-4Is [30]. Moreover, DPP-4Is increased the risk of dizziness compared to placebo [31]. To date there have been no systematic reviews of dizziness and headache after incretin-based therapies.

There are two potential mechanisms through which incretin-based therapies result in dizziness and headache. The first has to do with the impact on blood flow. Studies have shown that postprandial GLP-1 increases regional cerebral blood flow [32]. Moreover, population-based studies [33-37] have shown that GLP-1 RAs and DPP-4Is might have a role in reducing blood pressure, which may cause dizziness (see www.mayoclinic.org/diseases-conditions/dizziness/symptoms-causes/syc-20371787).

In addition, the dilation of cerebral blood vessels caused by lower blood pressure and increased regional cerebral blood flow may stretch surrounding nerves, causing them to send signals to the trigeminal system, which may cause headache (see www.scientificamerican.com/article/what-causes-headaches/). The second mechanism relates to the impact on neurological functions. Although GLP-1 RAs and DPP-4Is function differently, their ultimate purpose is the same: to extend the half-life of GLP-1 and increase its activity. GLP-1 RAs may act on the brain by passing through the blood-brain barrier as well as interacting with vagal afferent nerves [38]. DPP-4Is can block the enzyme DPP-4 and thereby increase levels of incretin. Similarly, GLP-1 influences various brain functions by interacting with afferent nerves of the autonomic nervous system, which distributes GLP-1 receptors [39-41]. Activation of certain areas of the human brain may cause these symptoms. However, more studies are needed to determine the exact relation between the agent and this kind of neurological manifestation.

As symptoms that affect the nervous system, headache and dizziness may have adverse impacts on the feelings, work, and lives of patients who take certain drugs and thereby may reduce compliance with a medication regimen. For example, a systematic review of patients with headache [42] showed that adherence rates range from 25% to 94%; this may affect disease control. Currently no clinical guidelines recommend how to deal with such symptoms and whether to change medication or reduce the dose.

The major advantage of our study is the use of NMA with high-quality studies to compare adverse events of dizziness and headache caused by incretin-based therapies and other antidiabetic drugs and placebo. Moreover, GRADE showed that the majority of the results were of high or moderate quality. Nevertheless, some limitations should be noted. Different studies had different standards for judging dizziness and headache. Investigators could only obtain subjective data reported by patients, which may have led to great uncertainty. Moreover, few studies indicated whether these symptoms affected patients’ compliance, extra medical burden, lifestyle, or psychological status. Another limitation is that the subgroup analysis and regression were based on the average result of each trial, which may not have accurately reflected every participant. Thus, readers should be cautious when using the results of this study to guide clinical practice.

### Conclusions

Incretin-based therapies increase the risk of dizziness and headache compared to insulin, TZD, and placebo, a fact that should be emphasized by physicians. Future guidelines should pay more attention to these therapies.

## Supplementary Materials

The Supplemenantry data can be found online at: www.aginganddisease.org/EN/10.14336/AD.2019.0303


