# Supplementary material for: Neurological Manifestation of Incretin-Based Therapies in Patients with Type 2 Diabetes: A Systematic Review and Network Meta-Analysis
Source: Aging Dis. 2019 Dec 1;10(6):1311–9. doi: 10.14336/AD.2019.0303 (PMC6844583; doi:10.14336/AD.2019.0303)
Supplement: Supplementary file 1 [file AD-10-6-1311-s.pdf]

SUPPLEMENTARY DATA

Neurological Manifestation of Incretin-Based Therapies in Patients with Type 2 Diabetes: A Systematic Review and Network Meta-Analysis

Le Gao[1*], Shuqing Yu[1], Andrea Cipriani[2], Shanshan Wu[3], Yi Huang[4], Zilu Zhang[5], Jun Yang[1], Yixin Sun[1], Zhirong Yang[6], Sanbao Chai[7], Yuan Zhang[8], Linong Ji[9], Siyan Zhan[1*], Feng Sun[1*]

[1]Department of Epidemiology and Biostatistics, School of Public Health, Peking University, Beijing, China. [2]Department of Psychiatry, University of Oxford, Oxford, OX3 7JX, UK. [3]National Clinical Research Center of Digestive Diseases, Beijing Friendship Hospital, Capital Medical University, Beijing, China. [4]Department of Mathematics and Statistics, University of Maryland Baltimore County, Baltimore, MD 21250, USA. [5]Harvard Medical School and Harvard Pilgrim Health Care Institute, Boston, MA 02215, USA. [6]Primary Care Unit, School of Clinical Medicine, University of Cambridge, Cambridge, CB1 8RN, UK. [7]Department of Endocrinology and Metabolism, Peking University International Hospital, Beijing, China. [8]Department of Clinical Epidemiology and Biostatistics, McMaster University, Hamilton, Ontario, Canada. [9]Department of Endocrinology and Metabolism, People's Hospital, Peking University, Beijing, China.

© 2019. Gao L et al. Published online at http://www.aginganddisease.org/EN/10.14336/AD.2019.0303

Supplementary Table 1. Search strategy for Embase.

Step	Search strategy
#1	'glucagon like peptide'/exp
#2	'glucagon like peptide 1'/exp
#3	'glp-1 receptor agonists'
#4	'glucagon like peptide 1 receptor agonists'
#5	'glucagon receptor'/exp
#6	'glucagon-like peptide-1 agonists'
#7	'glp-1 receptor agonist'
#8	'glucagon like peptide 1 receptor agonist'
#9	'glp-1 agonist'
#10	'glp-1 agonists'
#11	'glp-1 ra*'
#12	'exenatide'/exp
#13	'liraglutide'/exp
#14	'albiglutide'/exp
#15	'taspoglutide'/exp
#16	'lixisenatide'/exp
#17	'dulaglutide'/exp
#18	'semaglutide'/exp
#19	'zp10a peptide'
#20	'zp10a peptide 1'
#21	'glp1 ra'
#22	#1 OR #2 OR #3 OR #4 OR #5 OR #6 OR #7 OR #8 OR #9 OR #10 OR #11 OR #12 OR #13 OR #14 OR #15 OR #16 OR #17 OR #18 OR #19 OR #20 OR #21
#23	'dipeptidyl peptidase iv inhibitor'
#24	'dipeptidyl peptidase iv inhibitors'
#25	'dipeptidyl peptidase iv inhibitor'/exp
#26	'dpp 4 inhibitor*'
#27	'dpp iv inhibitor*'
#28	'dpp4i'
#29	'dpp4 i'
#30	'dpp4 inhibitor*'
#31	'dppiv inhibitor*'
#32	'alogliptin'/exp
#33	'sitagliptin'/exp
#34	'gemigliptin'/exp
#35	'linagliptin'/exp
#36	'saxagliptin'/exp
#37	'vildagliptin'/exp
#38	'dutogliptin'/exp
#39	'teneligliptin'/exp
#40	'anagliptin'/exp
#41	'trelagliptin'/exp
#42	'pf-734200'/exp
#43	'melogliptin'/exp
#44	'evogliptin'/exp
#45	'retagliptin'
#46	'carmegliptin'/exp
#47	'lc15 0444'
#48	'tenelia'
#49	'da-1229'
#50	'omarigliptin'/exp

© 2019. Gao L et al. Published online at http://www.aginganddisease.org/EN/10.14336/AD.2019.0303

#51	'beskoa'
#52	'gemiglo'
#53	'trajenta'
#54	'kazano'
#55	'oseni'
#56	'nesina'
#57	'kombiglyze xr'
#58	'onglyza'
#59	'eucreas'
#60	'galvus'
#61	'juvisync'
#62	'janumet'
#63	'januvia'
#64	'liptin'
#65	'gliptin'/exp
#66	#23 OR #24 OR #25 OR #26 OR #27 OR #28 OR #29 OR #30 OR #31 OR #32 OR #33 OR #34 OR #35 OR #36 OR #37 OR #38 OR #39 OR #40 OR #41 OR #42 OR #43 OR #44 OR #45 OR #46 OR #47 OR #48 OR #49 OR #50 OR #51 OR #52 OR #53 OR #54 OR #55 OR #56 OR #57 OR #58 OR #59 OR #60 OR #61 OR #62 OR #63 OR #64 OR #65
#67	#1 OR #2 OR #3 OR #4 OR #5 OR #6 OR #7 OR #8 OR #9 OR #10 OR #11 OR #12 OR #13 OR #14 OR #15 OR #16 OR #17 OR #18 OR #19 OR #20 OR #21 OR #22 OR #23 OR #24 OR #25 OR #26 OR #27 OR #28 OR #29 OR #30 OR #31 OR #32 OR #33 OR #34 OR #35 OR #36 OR #37 OR #38 OR #39 OR #40 OR #41 OR #42 OR #43 OR #44 OR #45 OR #46 OR #47 OR #48 OR #49 OR #50 OR #51 OR #52 OR #53 OR #54 OR #55 OR #56 OR #57 OR #58 OR #59 OR #60 OR #61 OR #62 OR #63 OR #64 OR #65 OR #66
#68	'clinical trial'/exp OR 'controlled clinical trial'/exp OR 'randomized controlled trial'/exp
#69	random*
#70	'case-control studies'/exp
#71	'retrospective studies'/exp
#72	'cohort studies'/exp
#73	'longitudinal studies'/exp
#74	'follow up studies'/exp
#75	nonrandom
#76	'prospective studies'/exp
#77	'comparison group'
#78	'control group'/exp
#79	database*
#80	'database'/exp
#81	'registries'/exp
#82	'registration'/exp
#83	#68 OR #69 OR #70 OR #71 OR #72 OR #73 OR #74 OR #75 OR #76 OR #77 OR #78 OR #79 OR #80 OR #81 OR #82
#84	#22 AND #83
#85	#22 AND #83 AND [humans]/lim
#86	#22 AND #83 AND [animals]/lim
#87	#86 NOT #85
#88	#84 NOT #87
#89	#66 AND #83
#90	#66 AND #83 AND [humans]/lim
#91	#66 AND #83 AND [animals]/lim
#92	#91 NOT #90
#93	#89 NOT #92
#94	#67 AND #83
#95	#67 AND #83 AND [humans]/lim

#96	#67 AND #83 AND [animals]/lim
#97	#96 NOT #95
#98	#94 NOT #97
#99	#88 AND ([article]/lim OR [article in press]/lim OR [conference abstract]/lim OR [conference paper]/lim)
#100	#93 AND ([article]/lim OR [article in press]/lim OR [conference abstract]/lim OR [conference paper]/lim)
#101	#98 AND ([article]/lim OR [article in press]/lim OR [conference abstract]/lim OR [conference paper]/lim)
#102	#99 AND [embase]/lim
#103	#100 AND [embase]/lim
#104	#101 AND [embase]/lim

Supplementary Table 2. Summary of characteristics of the 233 studies included in NMA.

Characteristics	Total (n=233)	Dizziness (n=141)	Headache (n=209)
**Trial duration (weeks)**	26 (24, 52)	24 (24, 52)	26 (24, 52)
**Sample size**	389 (240, 667)	389 (262, 689)	400 (259, 700)
**Background therapeutic modes (%)**			
None	66 (28.33)	41 (29.08)	59 (28.23)
Monotherapy	108 (46.35)	62 (43.97)	98 (46.89)
Combined treatment	59 (25.32)	38 (26.95)	52 (24.88)
**Age (years)**	56.32±5.12	55.90±4.66	56.03±4.83
**HbA1c (%)**	8.18±0.54	8.02±0.50	8.17±0.52
**Duration of diabetes (years)**	6.40 (4.50, 8.70)	6.35 (4.90, 8.80)	6.40 (4.45, 8.30)

Trial duration, sample size and duration of diabetes were expressed as median (interquartile range); Background therapeutic modes was expressed as n (%); Age and HbA1c were expressed as mean±SD.

Supplementary Table 3. Ranking probability.

Treatment	Dizziness		Headache	
	SUCRA	Rank	SUCRA	Rank
DPP-4Is	46.2	5	45.0	5
GLP-1 RAs	22.5	8	23.4	9
Insulin	83.5	2	80.6	2
Metformin	24.6	7	26.9	6
SGLT-2	97.9	1	78.3	3
Sulfonylurea	1.9	9	23.7	8
TZD	70.0	3	86.0	1
AGI	44.7	6	24.3	7
Placebo	58.8	4	61.8	4

DPP-4Is: dipeptidyl peptidase-4 inhibitors; GLP-1 RAs: glucagon-like peptide-1 receptor agonists; SGLT-2: sodium-glucose co-transporter 2; TZD: thiazolidinediones; AGI: alpha-glucosidase inhibitor.

© 2019. Gao L et al. Published online at http://www.aginganddisease.org/EN/10.14336/AD.2019.0303

SUPPLEMENTARY DATA

Supplementary Table 4. Quality of evidence using GRADE framework: Results of DPP-4Is and GLP-1RAs compared to other treatments.

Comparisons	Dizziness		Headache	
	**Confidence in OR**	**Reasons for downgrade**	**Confidence in OR**	**Reasons for downgrade**
DPP-4Is vs. GLP-1 RAs	High	-	High	-
DPP-4Is vs. Insulin	Moderate	Downgrade by one level due to study limitation	Moderate	Downgrade by one level due to study limitation
DPP-4Is vs. Metformin	High	-	Low	Downgrade by two levels due to heterogeneity and inconsistency, indirectness
DPP-4Is vs. SGLT-2	High	-	High	-
DPP-4Is vs. Sulfonylurea	Moderate	Downgrade by one level due to indirectness	High	-
DPP-4Is vs. TZD	Moderate	Downgrade by one level due to indirectness	High	-
DPP-4Is vs. AGI	Moderate	Downgrade by one level due to imprecision	Low	Downgrade by two levels due to study limitation and imprecision
DPP-4Is vs. Placebo	High	-	Moderate	Downgrade by one level due to indirectness
GLP-1 RAs vs. Insulin	Low	Downgrade by two levels due to study limitation	Low	Downgrade by two levels due to study limitation
GLP-1 RAs vs. Metformin	Moderate	Downgrade by one level due to imprecision	Very low	Downgrade by three levels due to imprecision, inconsistency and indirectness
GLP-1 RAs vs. Sulfonylurea	High	-	Moderate	Downgrade by one level due to inconsistency
GLP-1 RAs vs. TZD	Low	Downgrade by two levels due to indirectness and heterogeneity	Moderate	Downgrade by one level due to indirectness
GLP-1 RAs vs. Placebo	High	-	High	-
GLP-1 RAs vs. SGLT-2	High	-	High	-
GLP-1 RAs vs. AGI	Moderate	Downgrade by one level due to imprecision	Moderate	Downgrade by one level due to imprecision
Ranking of treatment	Low	Downgrade by two levels due to indirectness and publication bias	Moderate	Downgrade by one level due to indirectness

Imprecision was evaluated by using CINeMA (see http://cinema.ispm.ch/#imprecision) except for the ranking of treatment, the clinically important size of effect was defined from 0.8 to 1.25. We considered to give a downgrade only when the result was major concern, meanwhile we described the reasons for the downgrade. DPP-4Is: dipeptidyl peptidase-4 inhibitors; GLP-1 RAs: glucagon-like peptide-1 receptor agonists; SGLT-2: sodium-glucose co-transporter 2; TZD: thiazolidinediones; AGI: alpha-glucosidase inhibitor.

© 2019. Gao L et al. Published online at http://www.aginganddisease.org/EN/10.14336/AD.2019.0303

SUPPLEMENTARY DATA

Supplementary Table 5. Results of the inconsistency by node-splitting model.

Comparisons	Dizziness							Headache						
	Direct		Indirect		Difference			Direct		Indirect		Difference		
	Log OR	SE	Log OR	SE	Log OR	SE	P-value	Log OR	SE	Log OR	SE	Log OR	SE	P-value
DPP-4Is vs. Placebo	-0.07	0.05	-0.01	0.14	-0.06	0.15	0.703	-0.05	0.06	-0.15	0.10	0.10	0.12	0.374
DPP-4Is vs. GLP-1 RAs	0.15	0.17	0.30	0.09	-0.15	0.19	0.447	0.04	0.11	0.11	0.07	-0.08	0.13	0.549
DPP-4Is vs. Insulin	0.29	0.57	-0.44	0.18	0.73	0.60	0.223	0.19	0.40	-0.22	0.09	0.41	0.41	0.313
DPP-4Is vs. Metformin	0.25	0.21	0.34	0.43	-0.09	0.47	0.852	**-0.07**	**0.15**	**0.67**	**0.28**	**-0.74**	**0.33**	**0.023**
DPP-4Is vs. SGLT-2	-2.27	0.99	1.57	3.01	-3.84	3.05	0.208	-0.08	0.22	-1.33	0.66	1.25	0.71	0.079
DPP-4Is vs. Sulfonylurea	0.63	0.07	0.29	0.28	0.34	0.29	0.249	0.13	0.08	-0.04	0.14	0.17	0.17	0.294
DPP-4Is vs. TZD	-0.25	0.16	-0.06	0.21	-0.19	0.27	0.485	-0.37	0.13	0.04	0.20	-0.41	0.23	0.077
DPP-4Is vs. AGI	0.06	0.42	-0.07	602.05	0.13	602.05	1.000	1.11	1.64	-5.19	280.55	6.30	280.56	0.982
GLP-1 RAs vs. Placebo	-0.33	0.06	-0.35	0.15	0.02	0.16	0.886	-0.19	0.06	-0.10	0.10	-0.08	0.12	0.479
GLP-1 RAs vs. Insulin	-0.73	0.16	0.13	0.50	-0.86	0.52	0.097	-0.30	0.08	-0.01	0.35	-0.29	0.36	0.417
GLP-1 RAs vs. Metformin	0.06	0.42	-0.02	0.22	0.09	0.47	0.852	**0.51**	**0.23**	**-0.27**	**0.17**	**0.78**	**0.29**	**0.006**
GLP-1 RAs vs. Sulfonylurea	0.07	0.24	0.39	0.10	-0.31	0.26	0.234	-0.08	0.12	0.06	0.11	-0.15	0.16	0.366
GLP-1 RAs vs. TZD	-0.34	0.20	-0.54	0.17	0.20	0.26	0.446	-0.10	0.18	-0.51	0.15	0.41	0.23	0.070
Insulin vs. Placebo	1.44	1.20	0.30	0.17	1.15	1.20	0.341	0.40	0.46	0.11	0.09	0.28	0.47	0.546
Insulin vs. TZD	-0.33	0.63	0.26	0.21	-0.60	0.66	0.368	0.01	1.01	-0.05	0.14	0.07	1.02	0.949
Metformin vs. TZD	-	-	-	-	-	-	-	-0.57	0.36	-0.29	0.19	-0.29	0.41	0.480
SGLT-2 vs. Placebo	0.29	1.64	4.13	1.98	-3.84	3.04	0.208	0.68	0.34	-0.19	0.28	0.87	0.45	0.054
Sulfonylurea vs. Placebo	0.05	0.50	-0.70	0.08	0.75	0.51	0.138	-0.50	0.26	-0.13	0.08	-0.37	0.28	0.182
TZD vs. Placebo	0.49	0.53	0.09	0.14	0.40	0.55	0.466	-0.47	0.42	0.24	0.12	-0.71	0.44	0.107

The statistically significant results were shown in bold. DPP-4Is: dipeptidyl peptidase-4 inhibitors; GLP-1 RAs: glucagon-like peptide-1 receptor agonists; SGLT-2: sodium-glucose co-transporter 2; TZD: thiazolidinediones; AGI: alpha-glucosidase inhibitor.

Supplementary Table 6. P-value of node splitting model results in subgroup analysis.

Characteristics	DPP-4Is vs. Metformin	GLP-1 RAs vs. Metformin
**Total**	**0.023**	**0.006**
**Duration of T2DM**		
≤5 years	0.111	**0.041**
5-10 years	-	-
>10 years	-	-
**Mean HbA1c**		
≤7.5%	0.992	-
7.5%-8.0%	-	0.999
8.0%-8.5%	0.569	0.457
>8.5%	0.994	-
**Trial duration**		
≤24 weeks	0.999	-
24-48 weeks	0.811	0.164
>48 weeks	**0.022**	**0.022**

The comparisons with inconsistency were shown in bold. DPP-4Is: dipeptidyl peptidase-4 inhibitors; GLP-1 RAs: glucagon-like peptide-1 receptor agonists.

© 2019. Gao L et al. Published online at http://www.aginganddisease.org/EN/10.14336/AD.2019.0303

Supplementary Table 7. Confidence intervals and predictive intervals in subgroup NMA.

Characteristics	GLP-1 RAs vs. Placebo		Insulin vs. DPP-4Is		TZD vs. DPP-4Is	
	CIs	PrIs	CIs	PrIs	CIs	PrIs
**Total**	**(1.07, 1.31)**	**(0.94, 1.50)**	**(0.68, 0.98)**	**(0.62, 1.08)**	**(0.63, 0.96)**	**(0.57, 1.05)**
**Duration of T2DM**						
≤5 years	(0.83, 1.49)	(0.67, 1.85)	(0.61, 1.87)	(0.52, 2.16)	(0.53, 1.07)	(0.44, 1.31)
5-10 years	(1.08, 1.34)	(1.08, 1.34)	(0.68, 1.03)	(0.68, 1.03)	(0.60, 1.03)	(0.59, 1.03)
>10 years	(0.84, 1.83)	(0.52, 2.99)	(0.29, 1.36)	(0.20, 1.96)	-	-
**Mean HbA1c**						
≤7.5%	(0.89, 3.56)	(0.78, 4.03)	(0.26, 2.65)	(0.21, 3.26)	-	-
7.5%-8.0%	(1.06, 1.45)	(1.06, 1.45)	(0.59, 1.51)	(0.59, 1.53)	(0.34, 0.89)	(0.33, 0.91)
8.0%-8.5%	**(1.03, 1.36)**	**(0.98, 1.43)**	(0.66, 1.02)	(0.63, 1.06)	(0.68, 1.24)	(0.66, 1.28)
>8.5%	(1.00, 2.10)	(0.98, 2.13)	(0.63, 1.74)	(0.62, 1.77)	(0.53, 1.11)	(0.52, 1.13)
**Trial duration**						
≤24 weeks	(1.07, 1.47)	(1.07, 1.48)	(0.54, 1.24)	(0.54, 1.25)	(0.55, 1.06)	(0.55, 1.07)
24-48 weeks	(1.00, 1.66)	(0.71, 2.33)	(0.62, 1.27)	(0.46, 1.70)	(0.41, 1.07)	(0.32, 1.38)
>48 weeks	(0.92, 1.26)	(0.82, 1.40)	(0.58, 1.06)	(0.54, 1.14)	(0.65, 1.35)	(0.61, 1.44)

The comparisons which may be affected by estimated heterogeneity were bolded. DPP-4Is: dipeptidyl peptidase-4 inhibitors; GLP-1 RAs: glucagon-like peptide-1 receptor agonists; TZD: thiazolidinediones; CIs: confidence intervals; PrIs: predictive intervals.

Supplementary Table 8. Subgroup NMA: Results of DPP-4Is and GLP-1RAs compared to placebo.

Characteristics	Dizziness			Headache		
	No. of studies	DPP-4Is	GLP-1 RAs	No. of studies	DPP-4Is	GLP-1 RAs
**All trials**	141	1.07(0.97, 1.18)	**1.40(1.25,1.56)**	209	1.08(0.98,1.19)	**1.18(1.07,1.31)**
**Mean age**						
≤60 years	120	1.00(0.85,1.18)	**1.37(1.17,1.60)**	186	**1.12(1.01,1.25)**	**1.19(1.07,1.33)**
>60 years	20	1.11(0.98,1.27)	**1.41(1.17,1.70)**	22	0.91(0.78,1.06)	**1.24(1.01,1.52)**
Unclear	1	-	3.18(0.12,83.76)	1	-	0.31(0.01,8.27)
**Duration of T2DM**						
≤5 years	31	0.94(0.38,1.31)	1.27(0.82,1.98)	60	**1.23(1.01,1.49)**	1.11(0.83,1.49)
5-10 years	69	1.0(0.86,1.23)	**1.39(1.22,1.59)**	100	1.07(0.94,1.22)	**1.20(1.08,1.34)**
>10 years	19	1.09(0.95,1.25)	**1.56(1.05,2.31)**	23	1.25(0.75,2.09)	1.24(0.84,1.83)
Unclear	22	1.18(0.83,1.70)	1.22(0.56,2.63)	26	0.94(0.66,1.33)	1.15(0.58,2.28)
**Mean HbA1c**						
≤7.5%	10	0.95(0.39,2.34)	0.44(0.04,5.22)	16	1.53(0.81,2.89)	1.78(0.89,3.56)
7.5%-8.0%	37	1.09(0.96,1.23)	**1.47(1.25,1.73)**	54	0.91(0.80,1.04)	**1.24(1.06,1.45)**
8.0%-8.5%	56	1.03(0.83,1.27)	**1.24(1.03,1.50)**	84	1.06(0.90,1.25)	**1.19(1.03,1.36)**
>8.5%	30	1.11(0.84,1.47)	**1.65(1.10,2.49)**	42	**1.25(1.02,1.52)**	**1.45(1.00,2.10)**
Unclear	8	3.18(0.13,79.00)	1.08(0.29,4.00)	13	1.07(0.60,1.88)	1.11(0.42,2.93)
**Trial duration**						
≤24 weeks	73	1.06(0.86,1.31)	**1.36(1.12,1.65)**	98	1.10(0.93,1.30)	**1.26(1.07,1.47)**
24-48 weeks	22	0.86(0.54,1.36)	1.44(0.97,2.14)	47	1.05(0.81,1.36)	**1.29(1.00,1.66)**
>48 weeks	46	1.09(0.97,1.22)	**1.41(1.21,1.65)**	64	1.05(0.91,1.22)	1.07(0.92,1.26)
**Sample size**						
≤1000	127	1.04(0.88,1.21)	**1.36(1.17,1.59)**	187	1.11(0.99,1.25)	**1.17(1.05,1.30)**
>1000	14	1.10(0.97,1.25)	**1.37(1.14,1.65)**	22	1.04(0.86,1.25)	1.25(0.99,1.59)
**Sponsors**						
With industry	131	1.07(0.97,1.19)	**1.39(1.23,1.56)**	199	1.09(0.99,1.21)	**1.19(1.07,1.32)**
Unclear & without industry	10	0.79(0.16,3.79)	1.49(0.40,5.52)	10	0.78(0.39,1.58)	1.04(0.49,2.21)

The statistically significant results were bolded. DPP-4Is: dipeptidyl peptidase-4 inhibitors; GLP-1 RAs: glucagon-like peptide-1 receptor agonists.

© 2019. Gao L et al. Published online at http://www.aginganddisease.org/EN/10.14336/AD.2019.0303

SUPPLEMENTARY DATA

Supplementary Table 9. Meta-regression: Results of DPP-4Is and GLP-1RAs compared to placebo.

	Variables (No. of studies)	Comparison (No. of studies)	Dizziness		Headache	
			Proportional change in ORs (95% CIs)	P value	Proportional change in ORs (95% CIs)	P value
**Univariate meta-regression**	Age (128)	DPP-4Is	1.18(0.93,1.50)	0.178	0.86(0.70,1.05)	0.412
		GLP-1 RAs	1.15(0.76,1.74)	0.514	0.98(0.76,1.27)	0.901
	BMI (123)	DPP-4Is	0.99(0.92,1.06)	0.740	1.03(0.96,1.10)	0.447
		GLP-1 RAs	0.98(0.92,1.03)	0.426	0.97(0.93,1.02)	0.244
	HbA1c (124)	DPP-4Is	1.01(0.74,1.38)	0.942	0.15(0.95,1.38)	0.149
		GLP-1 RAs	0.98(0.70,1.37)	0.913	1.00(0.77,1.31)	0.979
	Duration of T2DM (111)	DPP-4Is	1.02(0.98,1.06)	0.334	**0.96(0.93,1.00)**	**0.027**
		GLP-1 RAs	1.02(0.97,1.07)	0.467	1.03(0.99,1.06)	0.162
**Multivariate meta-regression**	Age	DPP-4Is (111)	1.52(0.97,2.41)	0.069	1.25(0.81,1.94)	0.318
	BMI		0.99(0.92,1.07)	0.818	1.06(0.98,1.14)	0.177
	HbA1c		1.34(0.84,2.13)	0.219	1.17(0.94,1.46)	0.167
	Duration of T2DM		0.96(0.90,1.03)	0.315	0.95(0.89,1.00)	0.058
	Age	GLP-1 RAs (169)	1.13(0.57,2.24)	0.735	**0.67(0.45,1.00)**	**0.052**
	BMI		0.99(0.93,1.06)	0.786	0.95(0.91,1.00)	0.069
	HbA1c		1.01(0.67,1.54)	0.954	0.86(0.65,1.15)	0.316
	Duration of T2DM		1.01(0.93,1.11)	0.771	1.05(1.00,1.11)	0.057

The statistically significant results were bolded. The scale of age is per 10 years, BMI is per 1 kg/m^2, HbA1c is per 1%, duration of T2DM is per 1 year. DPP-4Is: dipeptidyl peptidase-4 inhibitors; GLP-1 RAs: glucagon-like peptide-1 receptor agonists.

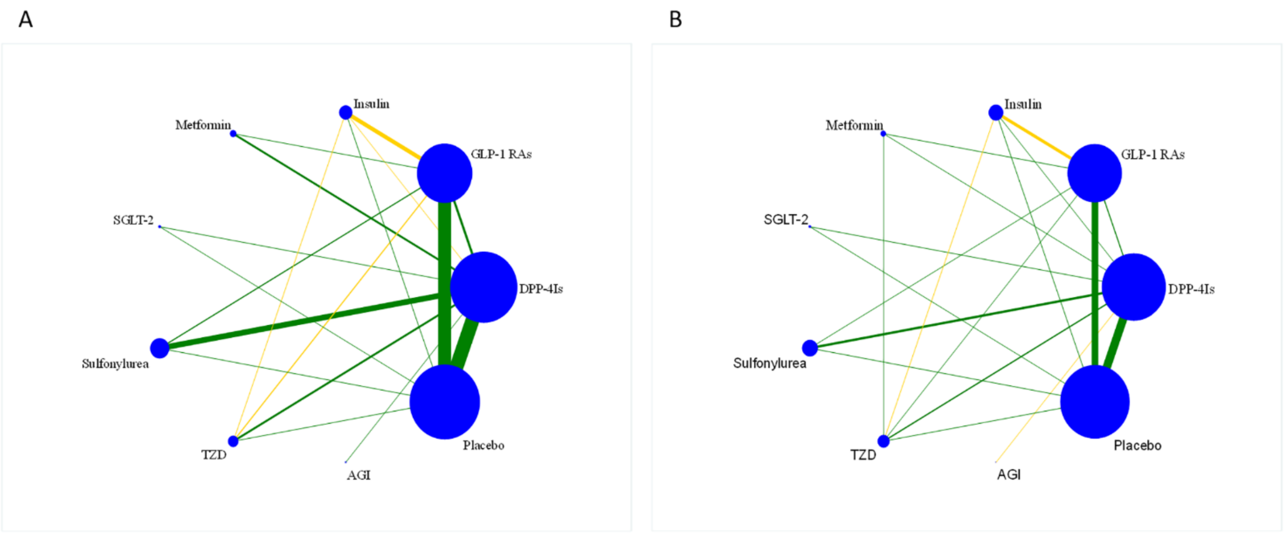

Supplementary Figure 1. Evidence structure of eligible comparisons for NMA: dizziness (A) and headache (B). Lines connect head-to-head (direct) comparisons in the eligible RCTs. The width of the lines represents the number of RCTs for each pairwise comparison and the size of each node is proportional to the number of randomized participants (sample size). The yellow lines represent trials with unclear risk of allocation concealment, which green represent low risk. DPP-4Is: dipeptidyl peptidase-4 inhibitors; GLP-1 RAs: glucagon-like peptide-1 receptor agonists; SGLT-2: sodium-glucose co-transporter 2; TZD: thiazolidinediones; AGI: alpha-glucosidase inhibitor.

© 2019. Gao L et al. Published online at http://www.aginganddisease.org/EN/10.14336/AD.2019.0303

SUPPLEMENTARY DATA

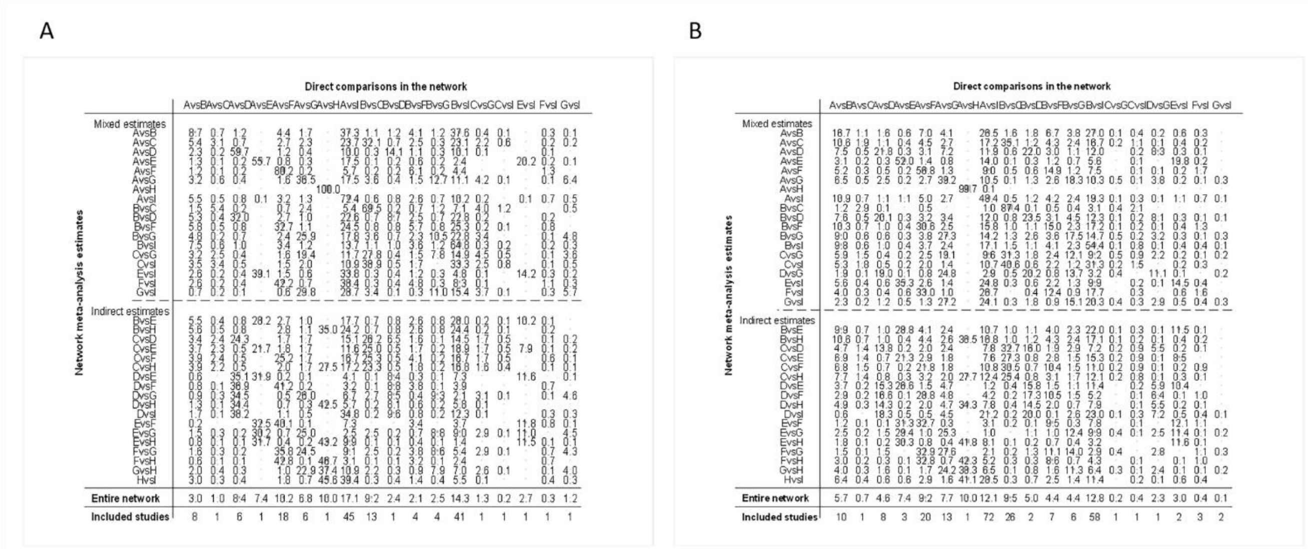

Supplementary Figure 2. Contribution plot for the incretin-based regimens network: dizziness (A) and headache (B). The size of the squares is proportional to the percentage contribution of the column-defining direct comparison to the row-defining network estimate. A= dipeptidyl peptidase-4 inhibitors, B= glucagon-like peptide-1 receptor agonists, C=insulin, D= metformin, E= sodium-glucose co-transporter 2, F= sulfonylurea, G= thiazolidinediones, H= alpha-glucosidase inhibitor, I=placebo.

© 2019. Gao L et al. Published online at http://www.aginganddisease.org/EN/10.14336/AD.2019.0303

SUPPLEMENTARY DATA

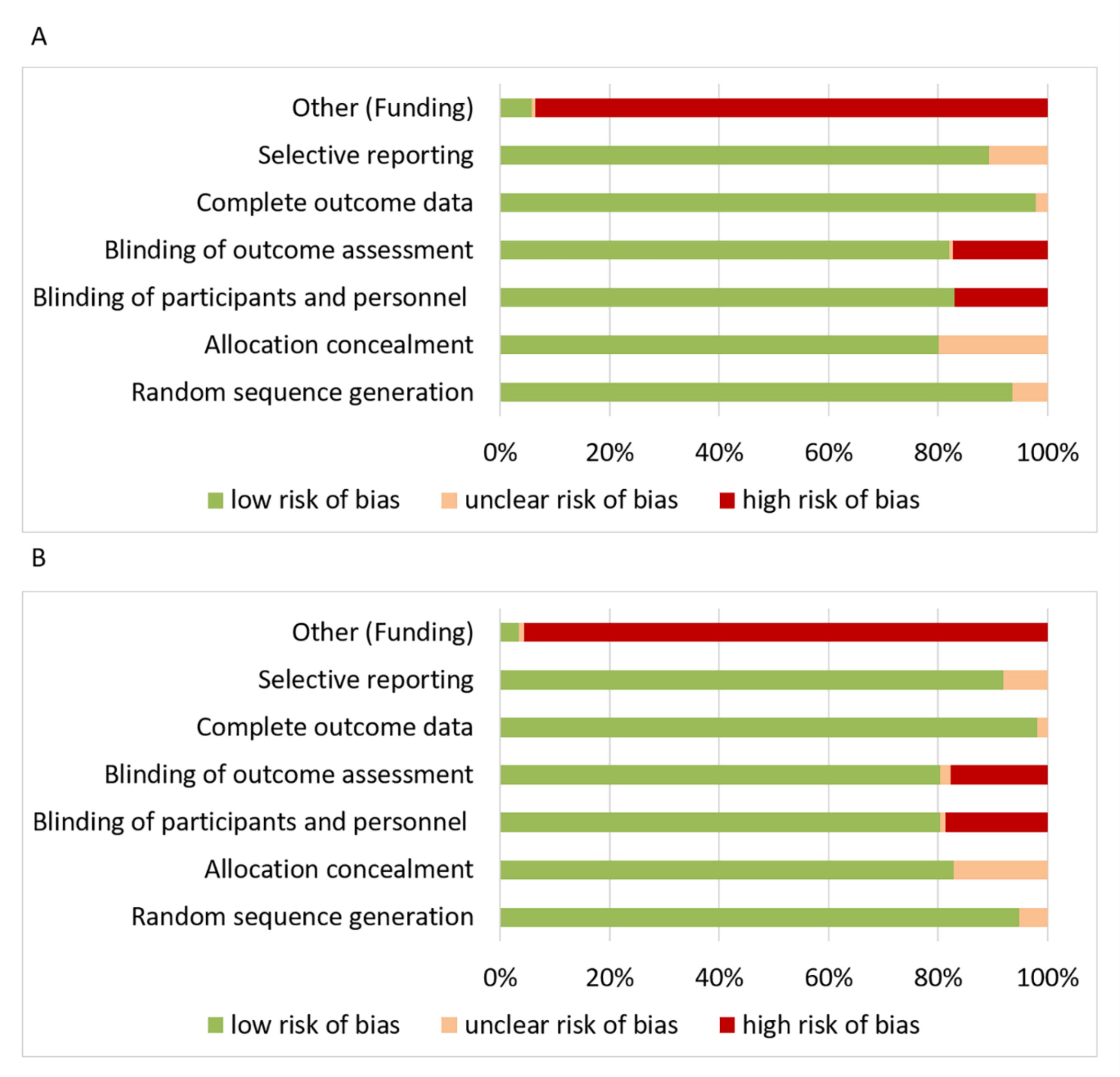

Supplementary Figure 3. Summary of risk of bias: dizziness (A) and headache (B).

© 2019. Gao L et al. Published online at http://www.aginganddisease.org/EN/10.14336/AD.2019.0303

SUPPLEMENTARY DATA

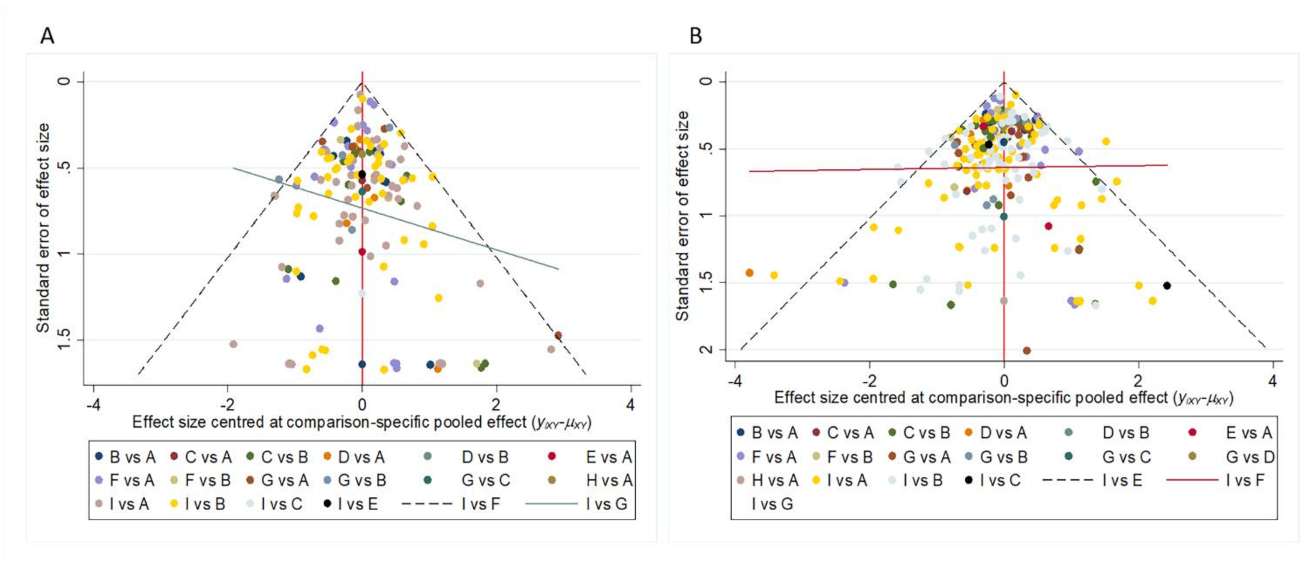

Supplementary Figure 4. Comparison-adjusted funnel plot: dizziness (A) and headache (B). A= dipeptidyl peptidase-4 inhibitors, B= glucagon-like peptide-1 receptor agonists, C=insulin, D= metformin, E= sodium-glucose co-transporter 2, F= sulfonylurea, G= thiazolidinediones, H= alpha-glucosidase inhibitor, I=placebo.

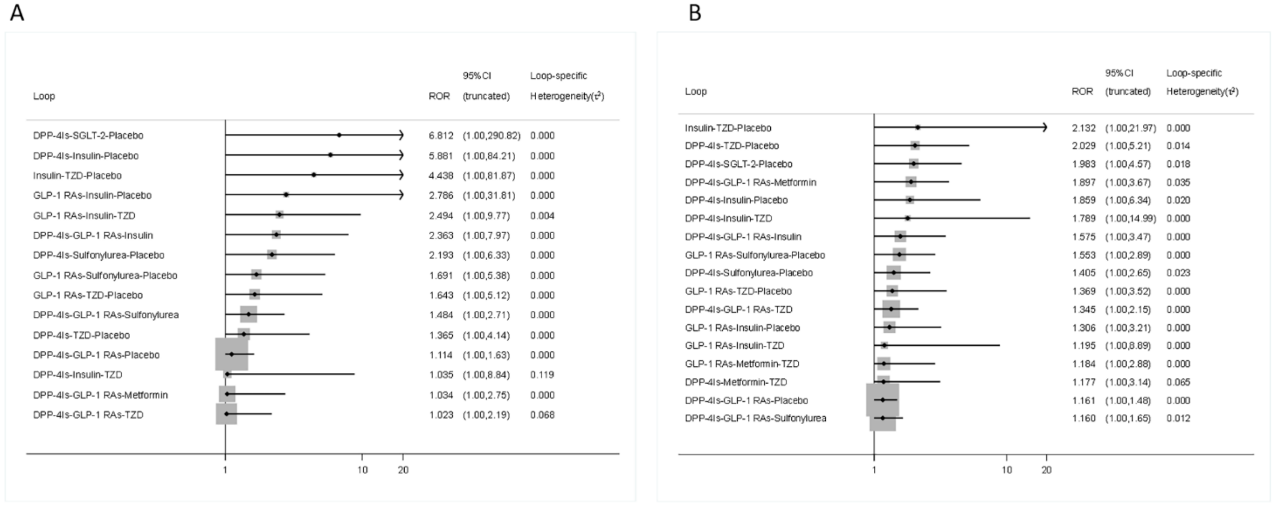

Supplementary Figure 5. Forest plots of inconsistency: dizziness (A) and headache (B). DPP-4Is: dipeptidyl peptidase-4 inhibitors; GLP-1 RAs: glucagon-like peptide-1 receptor agonists; SGLT-2: sodium-glucose co-transporter 2; TZD: thiazolidinediones.

© 2019. Gao L et al. Published online at http://www.aginganddisease.org/EN/10.14336/AD.2019.0303

SUPPLEMENTARY DATA

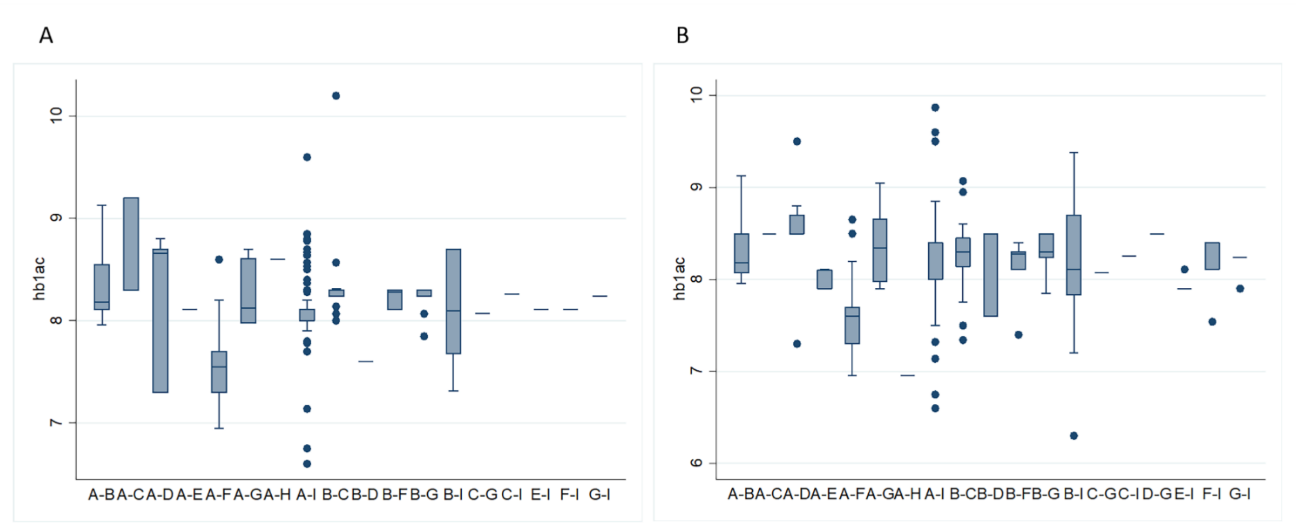

Supplementary Figure 6. Distribution of baseline HbA1c among different comparisons: dizziness (A) and headache (B). A= dipeptidyl peptidase-4 inhibitors, B= glucagon-like peptide-1 receptor agonists, C=insulin, D= metformin, E= sodium-glucose co-transporter 2, F= sulfonylurea, G= thiazolidinediones, H= alpha-glucosidase inhibitor, I=placebo.

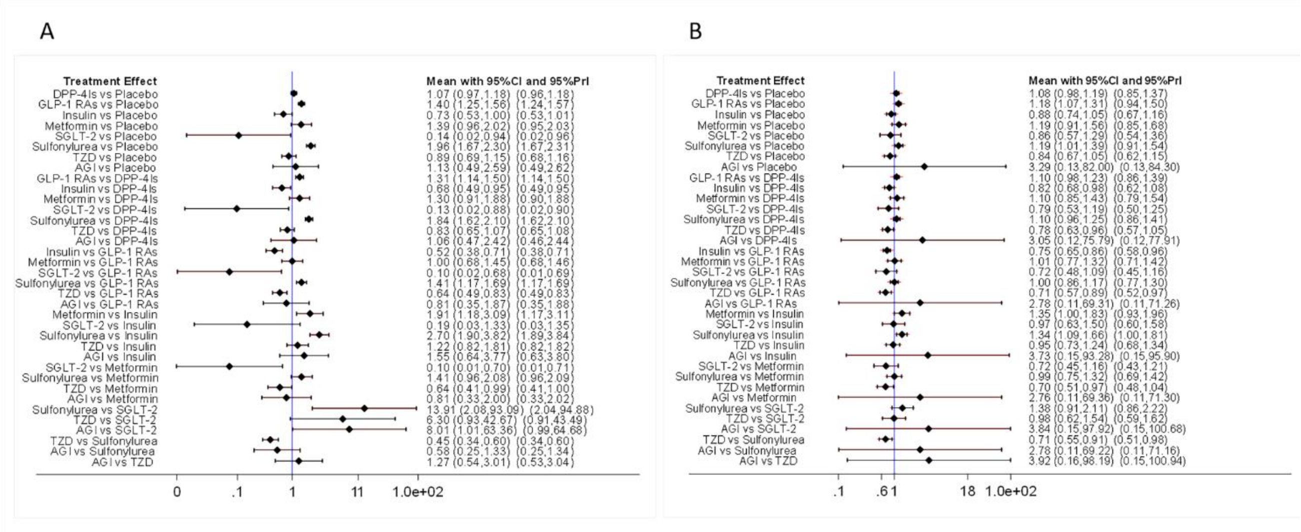

Supplementary Figure 7. Predictive interval plots: dizziness (A) and headache (B). The graph presents the network estimates for all pairwise comparisons. Black horizontal lines represent the confidence intervals, and red lines represent the predictive intervals. DPP-4Is: dipeptidyl peptidase-4 inhibitors; GLP-1 RAs: glucagon-like peptide-1 receptor agonists; SGLT-2: sodium-glucose co-transporter 2; TZD: thiazolidinediones; AGI: alpha-glucosidase inhibitor.

© 2019. Gao L et al. Published online at http://www.aginganddisease.org/EN/10.14336/AD.2019.0303

SUPPLEMENTARY DATA

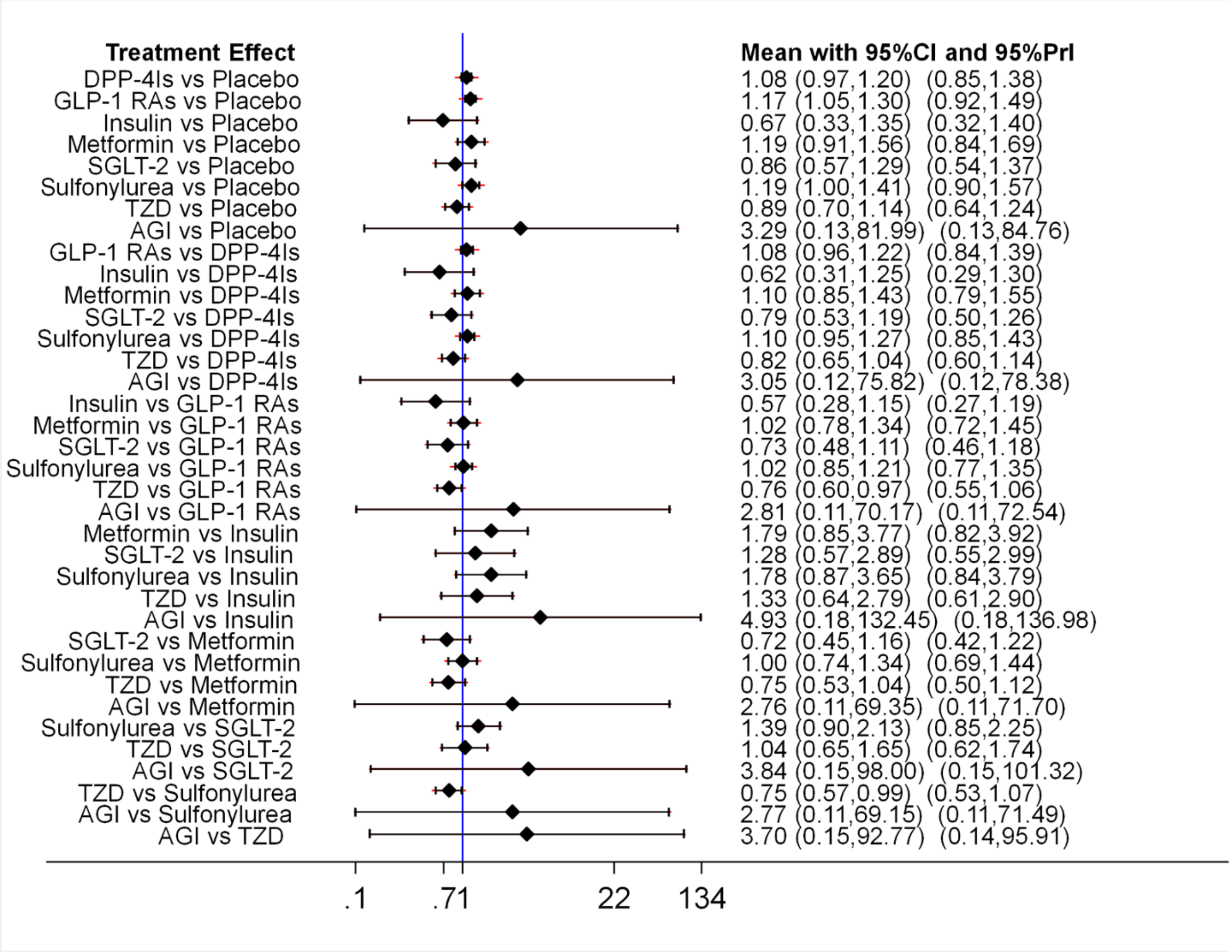

Supplementary Figure 8. Predictive interval plot of sensitivity analysis: headache. The graph presents the network estimates for all pairwise comparisons in sensitivity analysis. Black horizontal lines represent the confidence intervals, and red lines represent the predictive intervals. DPP-4Is: dipeptidyl peptidase-4 inhibitors; GLP-1 RAs: glucagon-like peptide-1 receptor agonists; SGLT-2: sodium-glucose co-transporter 2; TZD: thiazolidinediones; AGI: alpha-glucosidase inhibitor.

© 2019. Gao L et al. Published online at http://www.aginganddisease.org/EN/10.14336/AD.2019.0303

SUPPLEMENTARY DATA

A

**DPP-4Is**	0.92(0.64,1.34)	NA	0.78(0.52,1.17)	**12.58(1.28,123.37)**	**0.53(0.47,0.61)**	**1.66(1.12,2.47)**	0.94(0.41,2.15)	1.08(0.97,1.20)
**0.79(0.68,0.92)**	**GLP-1RAs**	3.05(0.32,29.57)	0.94(0.41,2.13)	NA	0.90(0.57,1.44)	1.22(0.62,2.38)	NA	**1.42(1.25,1.61)**
3.09(0.36,26.76)	3.89(0.45,33.61)	**Insulin**	NA	NA	NA	NA	NA	0.24(0.02,2.70)
0.77(0.53,1.12)	0.97(0.66,1.43)	0.25(0.03,2.23)	**Metformin**	NA	NA	NA	NA	NA
**7.55(1.13,50.46)**	**9.52(1.42,63.89)**	2.45(0.14,43.34)	**9.79(1.41,67.69)**	**SGLT-2**	NA	NA	NA	0.75(0.03,18.62)
**0.56(0.44,0.71)**	**0.70(0.53,0.93)**	0.18(0.02,1.58)	0.72(0.46,1.12)	**0.07(0.01,0.50)**	**Sulfonylurea**	NA	NA	0.92(0.32,2.61)
1.26(0.93,1.72)	**1.59(1.16,2.18)**	0.41(0.05,3.61)	**1.64(1.02,2.64)**	0.17(0.02,1.15)	**2.27(1.57,3.29)**	**TZD**	NA	0.61(0.21,1.76)
0.94(0.41,2.16)	1.19(0.51,2.76)	0.31(0.03,3.08)	1.22(0.49,3.03)	**0.12(0.02,0.99)**	1.70(0.71,4.03)	0.75(0.31,1.81)	**AGI**	NA
1.08(0.96,1.21)	**1.36(1.20,1.54)**	0.35(0.04,3.02)	1.40(0.96,2.04)	**0.14(0.02,0.96)**	**1.94(1.46,2.57)**	0.85(0.62,1.17)	1.15(0.49,2.65)	**Placebo**

B

**DPP-4Is**	0.90(0.72,1.12)	NA	1.15(0.71,1.84)	1.08(0.72,1.63)	0.94(0.83,1.07)	**1.39(1.06,1.83)**	0.33(0.01,8.11)	1.04(0.95,1.15)
0.92(0.82,1.04)	**GLP-1 RAs**	1.78(0.88,3.63)	**0.61(0.40,0.93)**	NA	0.94(0.72,1.23)	1.11(0.79,1.56)	NA	**1.25(1.13,1.39)**
1.62(0.80,3.28)	1.75(0.87,3.52)	**Insulin**	NA	NA	NA	NA	NA	0.69(0.29,1.67)
0.91(0.70,1.17)	0.98(0.75,1.29)	0.56(0.27,1.18)	**Metformin**	NA	NA	1.60(0.81,3.17)	NA	NA
1.26(0.84,1.89)	1.37(0.90,2.07)	0.78(0.35,1.75)	1.39(0.86,2.24)	**SGLT-2**	NA	NA	NA	**0.50(0.26,0.96)**
0.91(0.79,1.05)	0.98(0.83,1.17)	0.56(0.27,1.15)	1.00(0.75,1.35)	0.72(0.47,1.11)	**Sulfonylurea**	NA	NA	**1.78(1.03,3.07)**
1.21(0.96,1.54)	**1.31(1.03,1.68)**	0.75(0.36,1.57)	1.34(0.96,1.87)	0.96(0.60,1.53)	**1.33(1.01,1.76)**	**TZD**	NA	0.74(0.06,9.12)
0.33(0.01,8.16)	0.36(0.01,8.86)	0.20(0.01,5.45)	0.36(0.01,9.12)	0.26(0.01,6.64)	0.36(0.01,9.01)	0.27(0.01,6.79)	**AGI**	NA
1.08(0.97,1.20)	**1.17(1.05,1.30)**	0.67(0.33,1.35)	1.19(0.91,1.56)	0.86(0.57,1.29)	**1.19(1.00,1.41)**	0.89(0.70,1.14)	3.29(0.13,82.03)	**Placebo**

Supplementary Figure 9. Odds ratios (ORs) with 95%CIs of sensitivity analysis. For dizziness (A) and headache (B), results of direct comparisons were listed in the upper triangle, and the estimation was calculated as the row-defining treatment compared with the column-defining treatment. Results of NMA were listed in the lower triangle, and the estimation was calculated as the column-defining treatment compared with the row-defining treatment. The statistically significant results were bolded in red. NA: not available. DPP-4Is: dipeptidyl peptidase-4 inhibitors; GLP-1 RAs: glucagon-like peptide-1 receptor agonists; SGLT-2: sodium-glucose co-transporter 2; TZD: thiazolidinediones; AGI: alpha-glucosidase inhibitor.

© 2019. Gao L et al. Published online at http://www.aginganddisease.org/EN/10.14336/AD.2019.0303
